# Impact of COVID-19 early in the pandemic on the mental health and wellbeing of adolescents in Australia and Cambodia: a cross-national comparison using a quantitative descriptive and comparative approach

**DOI:** 10.3389/fpubh.2024.1360441

**Published:** 2024-07-23

**Authors:** Nina Van Dyke, Sam Oeun Keo, Maximilian P. de Courten

**Affiliations:** ^1^Mitchell Institute, Victoria University, Melbourne, VIC, Australia; ^2^Finn Church Aid, Phnom Pehn, Cambodia

**Keywords:** COVID-19, pandemic, mental health, wellbeing, adolescents, young people

## Abstract

The impact of COVID-19 on the mental health and wellbeing of adolescents is a major concern. Most research has been conducted only in more economically developed countries. Using data from two similar surveys administered during July–September, 2020 in Australia (a high-income country) and Cambodia (a low-middle income country), this paper examined the impact early in the pandemic on the mental health and wellbeing of adolescents in the two countries. We found that COVID-19 had mostly negative impacts on participants' mental health; threats to personal safety; education; support for schooling; basic necessities such as food, income, employment, and housing; and responsibilities at home. This finding suggests that even short-term disasters may have negative repercussions, and regardless of differences in wealth, culture, and government response. We found that threats to personal safety appeared to be more prevalent in Cambodia than in Australia, the impact on mental health of the Cambodian participants may have been greater than reported, and that, in both countries, support for online or distance schooling during periods of lockdown was wanting, particularly at the state and school levels. This study will contribute to our understanding of the impact of major disruptive global events on young people in both more economically developed and developing countries.

## 1 Introduction

COVID-19 was first detected in China in late 2019 and steadily escalated around the globe. In Australia, the first reported case was on 25 January 2020. In Cambodia, it was on 27 January 2020 (1). Once the World Health Organization (WHO) officially declared COVID-19 a global pandemic, many governments began to introduce emergency regulations to restrain the spread of the virus. These included travel restrictions, school closures, lockdowns, and social distancing. As a result of both fear of infection and restrictions, the impact of COVID-19 on the mental health of adolescents—both short-term and longer-term—is a major concern, as the adolescent years are a time when many mental health issues emerge (2). While an increasing volume of research has explored the impact of the pandemic on people's mental health and wellbeing [see, for example, Mansueto et al. (3), Alhakami et al. (4), and Brailovskaia et al. (5)], much of this work has been conducted only in more economically developed countries.

Using results from two similar surveys conducted during July–September, 2020 in Australia and Cambodia, this paper aimed to examine the early impact of the pandemic on the mental health and wellbeing of adolescents in the two countries. It is hoped that this analysis will contribute to our understanding of the impact of major disruptive global events on young people in both more economically developed and developing countries.

## 2 Background

### 2.1 Response to COVID: Australia and Cambodia

#### 2.1.1 Australia

Australia is classified as a high-income country according to The World Bank (https://data.worldbank.org). Australia suffered two major waves of COVID in 2020. The first, from February to July 2020, was mainly attributed to transmission by travelers to Australia from overseas. The second, from July to November 2020, was largely caused by breaches of hotel quarantine, which allowed the virus to spread to the community. By November 2020, Australia had effectively eliminated community transmission, which was mostly maintained in the first part of 2021 using snap lockdowns. These included border restrictions, social distancing, and shelter in place edicts. As a result, Australia had relatively low death rates compared to other economically developed countries. Short-term social and economic responses to the pandemic included increasing payments to the unemployed and the introduction of small and medium businesses loans. A National COVID-19 Coordination Committee was formed to oversee the COVID-19 Relief and Recovery Fund (6). A slow vaccination program, however, left Australia lagging behind comparable countries in terms of vaccination rates (7).

#### 2.1.2 Cambodia

Cambodia is classified as a lower middle-income country according to The World Bank (https://data.worldbank.org), but one that is described as blossoming economically. Between 1998 and 2019, it was one of the fastest-growing economies in the world and aspires to attain upper middle-income status by 2030 (https://www.worldbank.org/en/country/cambodia/overview#1).

Cambodia was relatively successful in containing the spread of the COVID-19 virus and limiting deaths early on during the pandemic. Reasons cited for this success included Cambodia's young population; its relatively recent experience with Severe Acute Respiratory Syndrome (SARS) and two rounds of the avian flu; its culture of wearing a face mask when sick; the government's vigorous implementation of contact tracing and strict quarantine; the effective mobilization of the military, government officials, and the Cambodian People's Party-affiliated youth associations for mass education to assist with food distribution, testing, quarantine, and vaccination campaigns; the government's ability to secure COVID-19 vaccines and its decisive implementation of mandatory vaccinations; significant support from international health partners; timely response to the virus of Cambodia's neighboring countries; and the compliance of the general public to the restrictions put in place (8–11).

The Cambodian government imposed state of emergency law and canceled the celebration of Khmer New Year and social gatherings (12). It established an inter-ministerial committee to combat COVID-19 and conducted a national communication campaign (13). It mandated that people wear masks, wash hands, maintain physical distance of at least 1.5 m, avoid confined and enclosed spaces, and avoid crowded spaces (14). It also introduced cash transfers to an estimated 560,000 poor and vulnerable households (14, 15). By the end of November 2021, the rate of vaccination among all eligible people in Cambodia, including children, was around 87 per cent (9).

**Figure 1:**
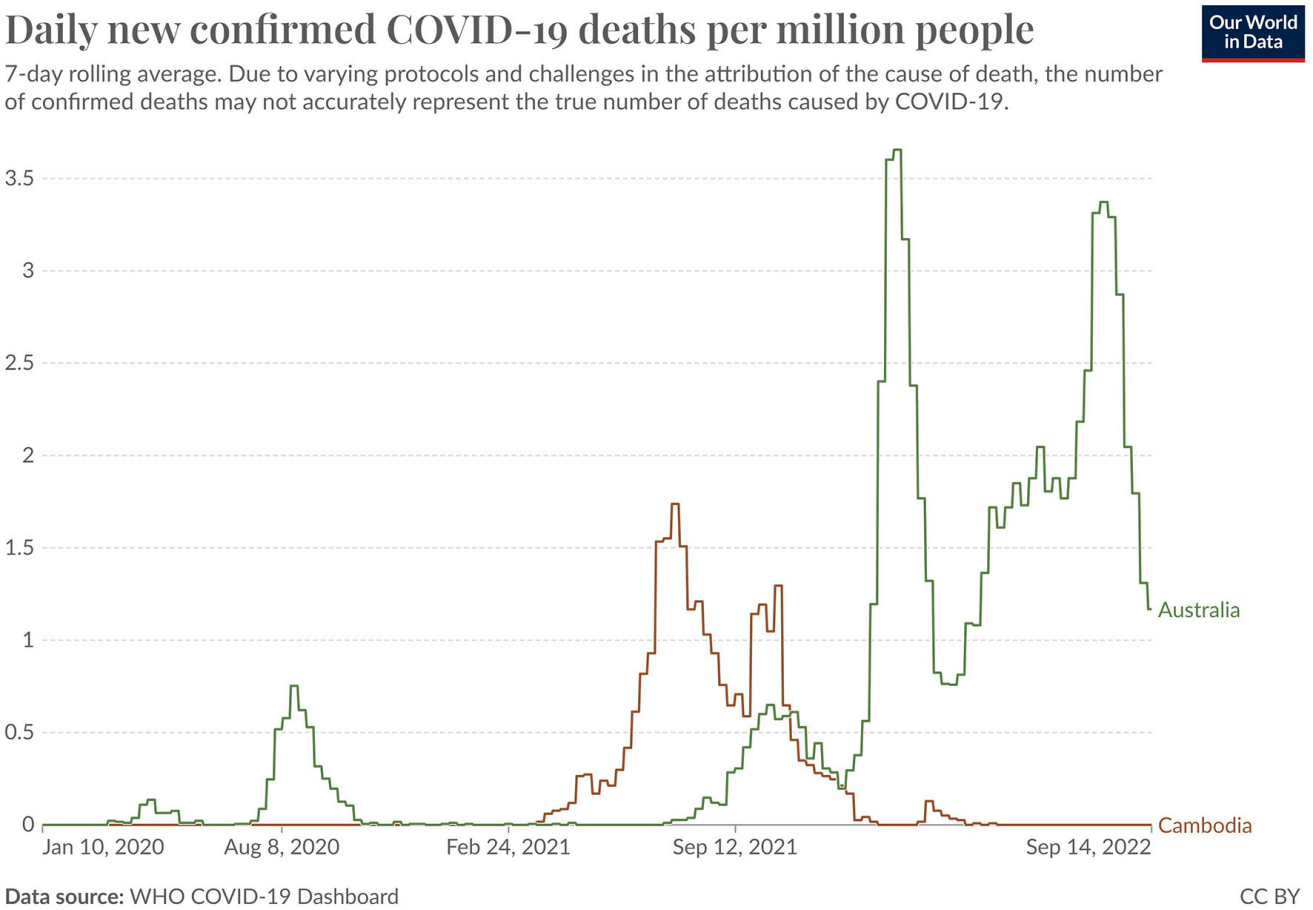
Daily new confirmed COVID-19 cases and deaths per million people: Australia and Cambodia (10 Jan 2020 – 14 Sep 2022). Reprinted with permission from Our World in Data, licensed under CC BY 4.0, https://ourworldindata.org/covid-deaths.

As can be seen in Figure 1, Australia as compared with Cambodia had earlier and higher rates of new cases, particularly starting from around December, 2021. Australia also had relatively early and mostly higher rates of new deaths, except between March and October, 2021 when rates in Cambodia were higher, and between October and December, 2021 when rates were similar.

**Figure 2:**
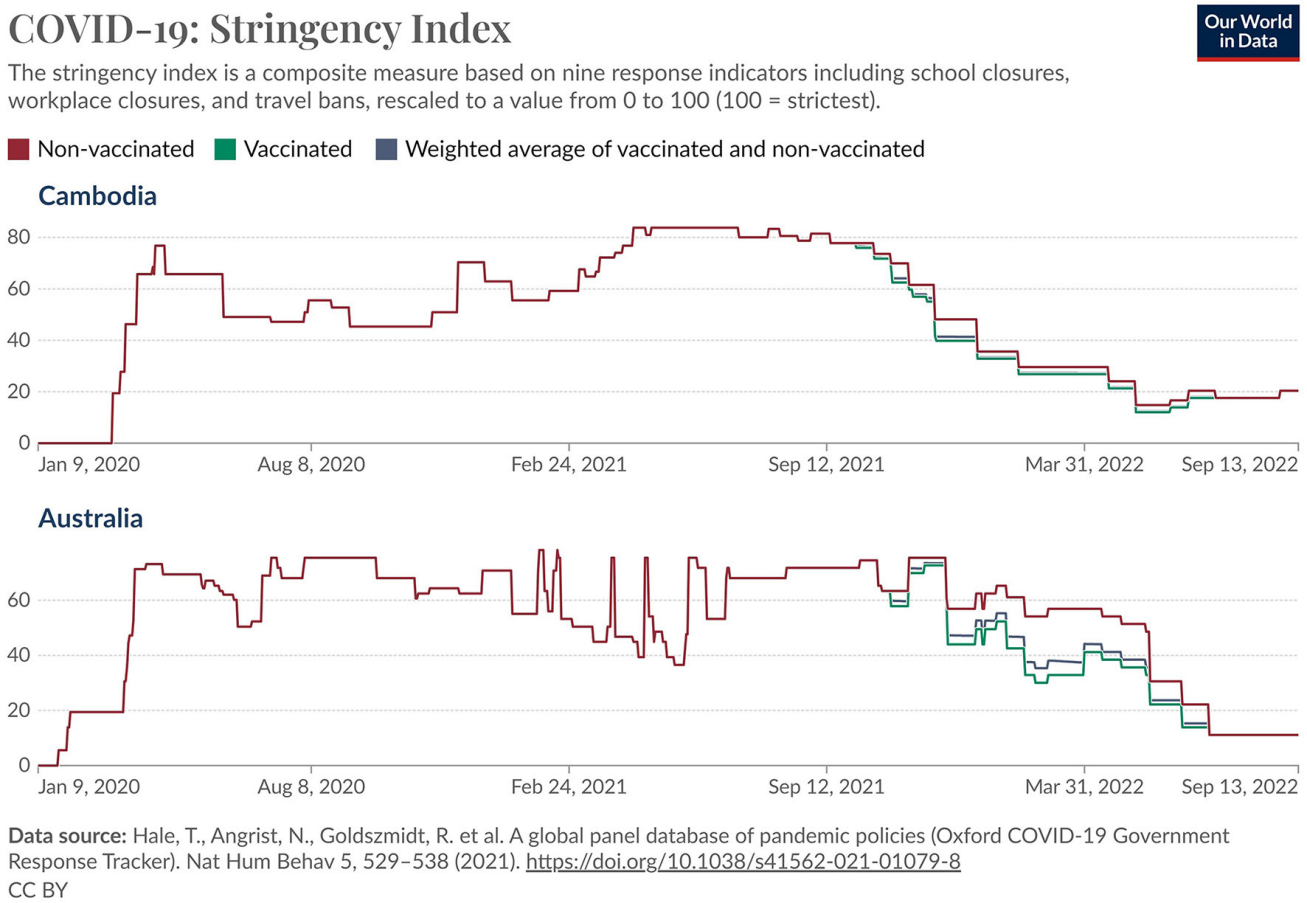
COVID-19 stringency index: Australia and Cambodia (9 Jan 2020 - 13 Sep 2022). Reprinted with permission from Our World in Data, licensed under CC BY 4.0, https://ourworldindata.org/covid-stringency-index.

Figure 2 shows level of stringency measures taken in response to the pandemic in each country over time. This measure combines school closures, workplace closures, cancellation of public events, restrictions on public gatherings, closures of public transport, stay-at-home requirements, public information campaigns, restrictions on internal movements, and international travel controls (16). The overall pattern was relatively similar in the two countries, with a rapid escalation in February–March, 2020, somewhat higher stringency levels in Australia between March, 2020 and November, 2021 followed by a decrease through May, 2022 but punctuated with large spikes, as compared with a constant increase in Cambodia over these same months, followed by a similar pattern in the two countries from May, 2022-on. This included a consistently high level through September, followed by a gradual decrease after that. It must be remembered, however, that stringency levels within Australia varied widely, with the State of Victoria in particular undergoing prolonged periods of lockdown and other restrictions. School closures lasted longer in Cambodia than in Australia.

### 2.2 Adolescents' mental health and wellbeing prior to the pandemic in Australia and Cambodia

#### 2.2.1 Australia

Results from the second Australian Child and Adolescent Survey of Mental Health and Wellbeing showed that 13.9% of Australian children and adolescents aged 4–17 had a mental disorder in the previous 12 months (17). Amongst adolescents aged 16–17, this rose to 14.7% (18). Rates of mental health and wellbeing were significantly lower for particular sub-groups within Australia, such as Indigenous adolescents (19).

#### 2.2.2 Cambodia

It is more difficult to obtain statistics on the mental health and wellbeing of adolescents in Cambodia. As noted in a 2017 PhD on the topic of the mental health and wellbeing of college students in Cambodia, most mental health research in Cambodia has been conducted on Cambodian refugees, older adult populations, and children with mental health disorders (20). Somasundaram (21) argued that young people aged 15–24 in post-conflict and transitional countries such as Cambodia are particularly vulnerable for high-risk behaviors and suicide. An earlier report by the Ministry of Education Youth and Sport Cambodia (22) found that 19% of young people aged 11–18 expressed suicidal thoughts and 14% had made suicidal plans. The general consensus appears to be that levels of mental health and wellbeing were generally lower in Cambodia than in a country such as Australia due to its relative higher rates of poverty and recent history of collective trauma (23, 24).

### 2.3 Impact of the pandemic on students' wellbeing in Australia and Cambodia

#### 2.3.1 Australia

Several Australian studies have examined the impact of COVID-19 on the mental health and wellbeing on adolescents. Most have found a negative impact, particularly for young people from already marginalized groups such as gender diverse, rural and remote, and Aboriginal and Torres Strait Islander, and those with a prior history of mental health issues (25–30). A few studies, however, have found a positive impact, mostly for students who experienced anxiety or bullying, and/or for whom remote learning was a better alternative to in-person learning (31–33).

#### 2.3.2 Cambodia

Compared with Australia, few Cambodian studies have examined the impact of COVID-19 on the mental health and wellbeing of adolescents. A study of families in Southwest Cambodia found that the pandemic resulted in financial insecurity, including less food, negative impacts on education through lack of access or the perceived need to work to support the other family members, increased risk and reduced safety through working on the streets or working at night, and increased vulnerability with less access to services and increased stress in the home (34). A survey on the impact of COVID-19 on school aged children and youth showed that their concern over the reduction in the quality of education was associated with poorer mental health and that adolescents were more likely to report feeling unsafe, resulting in discrimination, harassment, and violence or tensions within their community, during COVID-19 (35).

## 3 Methods

### 3.1 Data sets

For this research, we analyzed data from two surveys commissioned by UNICEF Australia and UNICEF Cambodia, respectively. Table 1 presents key information about the two data sets analyzed. The Australian survey was conducted online between 9 July and 4 August 2020 by YouGov Galaxy (now YouGov), a survey research company (https://au.yougov.com/). It consisted of a national, statistically representative sample of 1,289 young people aged between 13 and 17 years drawn from its youth panel (36). The data were weighted by age, gender, and region to reflect the latest Australian Bureau of Statistics population estimates (37). The questionnaire was co-developed by the UNICEF-Australia researchers and the YouGov Technical Research Team. The survey was piloted and modified as needed.

**Table 1:** Key information about surveys.

	**Australian survey**	**Cambodian survey**
Population	National; ages 13–17	National; years 7–12, ages 12–21
Date surveyed	9 July−4 August 2020	17 August−14 September 2020
Sample size	1,289	3,013
Mode	Online	Face-to-face, mobile, and online

The Cambodian survey was conducted between 17 August and 14 September, 2020. It consisted of a sample of 3,013 Cambodian students in Grades 7–12, aged 12–21. It used a multi-stage cluster sampling methodology, with clusters selected based on purposive selection of certain representative provinces and districts (rural/urban/disadvantaged area), with participants from each cluster school chosen based on variables including sex and grade level (38). Students completed the survey at their respective schools. The questionnaire was co-developed by the UNICEF-Cambodia researchers and MoEYS. The questions were approved by the technical group working on the study, which included both the Ministry and development partner representation. The survey was piloted and modified as needed.

### 3.2 Ethics approval

The UNICEF-Cambodia survey received ethics approval from the HLM IRB (602CAMB22). The UNICEF-Australia did not go through a formal Human Research Ethics procedure. However, all UNICEF commissioned surveys must adhere to the UNICEF Procedures for Ethical Standards in Research, Evaluation, Data collection, and Analysis (39). In both countries, informed consent from participants was obtained. No identifying information was included in either data set used for this analysis (40).

### 3.3 Items included in analyses

The items from each survey included in this study are presented in Table 2. They fall into five themes: mental health; threats to personal safety; impact on education; support for schooling; income/work/housing issues; and increased responsibilities at home.

**Table 2:** Survey items.

**Theme**	**Questions—Australian survey**	**Questions—Cambodian survey**
Mental health	How would you rate your overall ability to cope at the following times?—At the end of January 2020, before COVID-19 *(5-point scale)*	Have you displayed signs of mental health distress as a result of being out of school or as a result of the COVID-19 pandemic? Select all that apply: (*Tired; Frustrated; lonely; Sad, scared, nervous, agitated, angry, all, none, DK)*
	How would you rate your overall ability to cope at the following times?—Right now (July 2020) *(5-point scale)*	
	To date, how has the COVID-19 pandemic and government responses impacted on the following for you?—*Your level of stress and anxiety; your level of hope; your social connectedness (5-point scale)*	
	What are the things you're most worried about in relation to the pandemic and responses. I am worried about…? (Please choose up to 5)—*Being isolated from my friends and schoolmates*	
	Which of the following things have you experienced since Australia started to respond to the pandemic? Choose any that apply to you: *I have had to stop seeing my friends*	
Threats to personal safety	What are the things you're most worried about in relation to the pandemic and responses. I am worried about…? *(Please choose up to 5)—Threats to my personal safety being increased because of the effects of the pandemic and response measures*	Compared to the time before COVID-19, have you experienced an increased threat in any of the following protection issues as a result of being out of school?
		*Select all that apply: Violent discipline/physical abuse; Sexual abuse or exploitation; emotional abuse (e.g. neglect); Child marriage; Risk of juvenile offense (i.e. associations with gangs, substance abuse, stealing, viewing pornographic material); Receiving a sexual message, inappropriate image, or video; Child labor; None of the above*
Impact on education	Which of the following things have you experienced since Australia started to respond to the pandemic? *Choose any that apply to you: My education had been disrupted or stopped entirely*	Have you dropped out or are at risk of dropping-out if the schools remain closed for a prolonged time?
	What are the things you're most worried about in relation to the pandemic and responses. I am worried about…? *(Please choose up to 5)—My education being disrupted/held back*
	What effect do you think Australia's response to the pandemic, including school closures, has had on your schooling? *(no effect; behind; ahead)*
Support for schooling	*[If said behind compared to before COVID]* How much support are you receiving/ have you received from your school to catch up with your schooling*? (A lot; Some; None)*	Are your caregivers available and able to support your learning at home? *(All of the time; often; sometimes; never)*
	Do you receive additional education support after school? *(yes—school/teacher; yes—parent/carer; yes—paid tutor/tuition class; no)*	Do you think that the support/guidance you have received to access/ engage in distance learning is adequate? *(Completely inadequate; inadequate; neither; adequate; completely adequate)*
Access to basic necessities (Income/work/employment housing/food access issues)	What are the things you're most worried about in relation to the pandemic and responses. I am worried about…? *(Please choose up to 5)—Losing our family's income through job loss; losing my housing; My own income and employment being cut back or stopped*	Have you or anyone in your household received financial support from the government as part of the COVID19 cash assistance scheme, or through other private sources? *(Yes—from government's cash scheme; Yes—from a private sector cash scheme; Yes—from a local NBO/INGO cash scheme; no)*
	Which of the following things have you experienced since Australia started to respond to the pandemic? *Choose any that apply to you: My parent/s or carer has lost all or part of their income; I have lost my housing/accommodation*	Has your family experienced any change in access to food during school closure? *Select one (more, same, less, DK)*
Increased responsibilities at home	How has the COVID-19 pandemic and responses affected your responsibilities at home? *(Choose all that apply): I do more cooking at home; I do more household maintenance (for example, gardening, yard work, putting rubbish out); I do more home cleaning now (for example, tidying up, clothes washing, washing dishes/load-unload dishwasher, vacuuming floors); I look after/supervise my sibling/s more—including schoolwork and babysitting; I have more caring responsibilities for my extended family (including grandparents); I feel a responsibility to pick up as much paid work as I can to help my family income; I am now the main breadwinner in my family; My responsibilities have generally decreased; My responsibilities have not really changed*	Have you started working/contributing to more household chores in order to mitigate the effects of household income decline during COVID-19? *Select one (yes, working FT; yes, working PT; Yes, children are required to contribute more to HH chores; No)*

### 3.4 Analysis

Given that the surveys ask different questions, albeit on similar topics, it is impossible to directly compare responses. Therefore, we have chosen to use a descriptive and comparative approach to analysis. In so doing, we follow the advice of Herndon (41), who outlines key considerations in comparing results from different surveys and survey questions:

- Is the primary purpose of the surveys the same/similar?
- Are the populations the same/similar?
- How similar are the survey methodologies?
- How similar/different is the question-wording?

The primary purposes of the UNICEF–Australia and UNICEF–Cambodia surveys were similar but not identical. The primary purpose of the UNICEF–Australia survey was to understand the views and lived experiences of young people in Australia early on during the COVID-19 pandemic and the government response to it. This survey was part of phase two of a three-phase project. Phase one took place in January, 2020. The primary purpose of the UNICEF–Cambodia survey was to conduct a rapid needs assessment of the education sector. It included surveys of teachers, school directors, education administrators, teacher educators, and teacher trainees, as well as students. The needs assessment had three key objectives: (1) to understand to what extent COVID-19 impacted on the education system, and the health, safety and social protection of the target groups; (2) to measure and evaluate distance learning accessibility, quality, and effectiveness; and (3) to investigate educational outreach and learning activities undertaken by schools and teacher education colleges/institutions and to identify the capacity needs of educators to support distance learning at both national and subnational levels (38).

The populations of the two surveys analyzed were almost identical. Both were national surveys; the population of the UNICEF–Australia survey was ages 13–17 (36) the population of the UNICEF–Cambodia survey was years 7–12, which equates to approximately age 12–21 (38).

The survey methodologies, however, differed. The UNICEF-Australia survey invited participants to respond to the survey from the YouGov panel of young people, who were also paid a small amount to complete the survey. The sample was intended to be representative on key characteristics of the population of young people in Australia, but this goal was not achieved. All participants completed the survey online. The UNICEF-Cambodia survey used multi-stage cluster sampling of schools, with clusters based on purposive selection of certain representative provinces and districts (rural/urban/disadvantaged area), with participants from each cluster school based on variables such as gender and grade level. Participants completed the survey via in-person mobile (42). In both cases, individual young people were the participants. The question-wording of the two surveys was mostly quite different. These differences (and similarities) are discussed in the Results and Discussion sections.

Our conclusion was that the primary purpose of the two surveys was similar, and the populations were essentially identical; the methodologies and question-wordings, however, were different, and these differences must be considered in discussing the findings. Another important consideration not identified by Herndon (41) but of particular relevance for this study was under which COVID-19 related conditions the two surveys were conducted. As indicated above, both surveys were conducted during similar months in mid-2020, but for each country, the COVID-19 impact and government reactions were somewhat different.

## 4 Results

### 4.1 Mental health

It is clear from the data that the pandemic and government responses to it negatively affected the mental health of large percentages of young people in both Cambodia and Australia (refer to Tables 3–7). Looking at the most directly comparable data, 37% of Australian participants said the pandemic negatively affected their stress and anxiety levels, 26% their level of hope, and 43% their social connectedness; this compared with 21% of Cambodian students who said they felt frustrated, 16% who said they felt agitated, and 28% who said they felt lonely as a result of COVID-19 or being out of school.

**Table 3:** Overall ability to cope before COVID-19 and currently (Australia).

	***N* (Poor/very poor)**	**%**	**Chg. in %**
Overall ability to cope before COVID-19 (Jan 2020)	102	7.9	
Overall ability to cope right now (July 2020)	177	13.7	+73.5

**Table 4:** Impact of COVID-19 pandemic and government responses (Australia).

	**% Negatively**	**% Neutral**	**% Positively**
Stress and anxiety	37.0	38.0	24.9
Hope	26.2	38.1	35.7
Social connectedness	43.3	27.5	29.1

**Table 5:** Things most worried about in relation to the pandemic and responses (Australia).

	***N***	**%**
Being isolated from my friends and schoolmates	604	46.9

**Table 6:** Experienced since Australia started to respond to the pandemic (Australia).

	***N***	**%**
I had to stop seeing my friends	837	64.9

**Table 7:** Signs of mental health distress as a result of the COVID-19 pandemic or being out of school (Cambodia).

	**Frequency**	**%**
Sad	784	32.1
Lonely	659	27.9
Scared	541	23.9
Tired	476	21.7
Frustrated	440	20.5
Agitated	306	15.9
Angry	203	12.4
Nervous	106	9.2
None of the above	981	33.3

Australian participants who said their ability to cope was poor or very poor increased by 74% when asked about before COVID-19 and compared with currently. Almost half said their level of social connectedness had been negatively impacted (43%), and that being isolated from friends and schoolmates was one of their greatest worries about the impact of COVID-19 (47%). Two-thirds (67%) of Cambodian participants said that the COVID-19 pandemic had increased at least one of the eight indicators of mental health distress.

It should be noted, however, that the pandemic did not necessarily worsen mental health for all young people. Amongst Australian participants, about one quarter said the pandemic had positively impacted on their level of stress and anxiety (25%) and social connectedness (29%). More Australian participants said the pandemic had impacted their level of hope positively than negatively (36% vs. 26%), although it is possible that participants responded to this question in reference to getting past the initial impacts of COVID-19—that they had more hope currently than they did at the start of the pandemic. From the Cambodian data, we see that one-third of participants (33%) said the pandemic did not increase any of the eight indicators of mental health distress, although, due to the question wording, we do not know if there were decreases.

### 4.2 Threats to personal safety

On the face of it, it appeared that personal threats as a result of COVID-19 were a larger issue for Australian students (13.9%) as compared with Cambodian students (1.2%−5.6%; refer to Tables 8, 9). The questions, however, were somewhat different between the two countries. Although both surveys asked respondents about threats to personal safety, the Cambodian questions were more specific (i.e., they specified the type of threat) and, arguably, more serious in nature (e.g., it is unlikely that the Australian respondents were referring to child marriage or child labor when they answered the question). Although it is unknown what types of threats to personal safety Australian respondents had in mind when answering this question, on face value it appeared that the Cambodian questions about violent discipline/physical abuse, emotional abuse, and sexual abuse or exploitation were the most comparable. These responses were chosen by 2.7%, 3.8%, and 1.2% of the Cambodian sample, respectively.

**Table 8:** Worried about in relation to the pandemic and responses (Australia).

	***N***	**%**
Threats to my personal safety increased	179	13.9

**Table 9:** Compared to the time before COVID-19 experiences of increased threat as a result of being out of school (Cambodia).

	***N***	**%**
Violent discipline/physical abuse	77	2.7
Sexual abuse or exploitation	34	1.2
Emotional abuse (e.g. neglect)	107	3.8
Child marriage	159	5.6
Risk of juvenile offense (i.e. associations with gangs, substance abuse, stealing, viewing pornographic material)	102	3.6
Receiving a sexual message, inappropriate image, or video	40	1.4
Child labor	160	5.6
**“Yes” to at least one statement**	**679**	**23.9**

Whereas the Cambodian question asked, “have you experienced,” the Australian question asked, “things you're most worried about.” One would expect higher frequencies for worries than for personal experience. The Cambodian questions also asked about each threat separately, whereas the Australian question asked about threats overall (but with a limit on choosing up to five from the list of 18 options provided). Further examination of the data revealed that 23.9% of Cambodian students responded “yes” to at least one of the statements and 7.7% to the most comparable statements. Another difference between the two questions was that the Cambodian question asked about threat “as a result of being out of school,” whereas the Australian question asked about “in relation to the pandemic and responses,” which may or may not evoke the same responses.

Therefore, it appears that the impact of the pandemic on personal safety may have been greater and/or more serious in Cambodia than it was in Australia.

### 4.3 Education

The data indicated that the COVID-19 pandemic negatively impacted the education of both Australian and Cambodian survey participants.

Whereas the Cambodian participants were asked about risk of dropping out of school, Australian participants were asked about their education being disrupted or of falling behind (refer to Tables 10–13). The data showed that just under 12% of the Cambodian participants said they were at some risk of dropping out of school if schools remained closed for a prolonged time. The Australian data showed that large percentages of participants said they had experienced disruption to their education as a result of the pandemic (62%), believed they were now behind in their schooling (55%), and were worried about their education being disrupted or being held back as a result (46%). Thirteen percent of Australian participants, however, said they felt they were ahead in their schooling as a result of COVID-19 and school closures.

**Table 10:** Education disrupted or stopped entirely since Australia started to respond to the pandemic (Australia).

	**N**	**%**
My education has been disrupted or stopped entirely	797	61.8

**Table 11:** Things most worried about in relation to pandemic and responses (Australia).

	***N***	**%**
My education being disrupted/held back	593	46.0

**Table 12:** Effect of Australia's response to the pandemic including school closures, on your schooling (Australia).

	**% behind**	**% no effect**	**% ahead**
Effect of Australia's response to the pandemic, including school closures, on schooling	55.2	32.1	12.7

**Table 13:** Dropped out or at risk of dropping out if schools remained closed for a prolonged time (Cambodia).

	***N***	**%**
Yes, already dropped out	20	0.7
Not yet, but high risk	174	5.9
Not yet, but medium risk	47	1.6
Not yet, but low risk	104	3.5
**Total—any risk**	**345**	**11.7**
Would not drop out	2,598	88.3

### 4.4 Support for schooling

It appeared from the data that support for online or distance schooling during periods of school shutdowns was considered inadequate by large percentages of survey participants in both Cambodia and Australia—particularly support from schools or the education system, as compared with support from parents or carers (refer to Tables 14–17). More than half (53%) of Cambodian students, for example, said that the support or guidance they received was inadequate or completely inadequate, and more than three-quarters of Australian students said they received either no help from school to catch up with schooling (13%) or some help (64%).

**Table 14:** (If said behind) Amount of support received from school to catch up with schooling (Australia).

	***N***	**A lot**	**Some**	**None**
*[If said behind]* Amount of support from school to catch up with schooling	711	22.8	63.9	13.4

**Table 15:** Whether receive additional education support after school (Australia).

	***N***	**%**
Receive additional education support after school from school/teacher	255	19.8
Receive additional education support after school from parent/carer	434	33.7
Receive additional education support after school from paid tutor/tuition class	158	12.3
Do not receive additional education support after school	614	47.6

**Table 16:** Caregivers available and able to support learning at home (Cambodia).

	***N***	**%**
All of the time	846	28.9
Often	767	26.2
Sometimes	1,135	38.7
Never	184	6.3

**Table 17:** Whether support/guidance received to access/engage in distance learning is adequate (Cambodia).

	***N***	**%**
Inadequate or completely inadequate	1,541	53.1
Neither adequate nor inadequate	717	24.7
Adequate or completely adequate	645	22.2

Whereas just one-fifth (19%) of Australian students said they had received additional education support after school from the school or a teacher, one-third (34%) said they had received such support from a parent or carer (an additional 48% said they had received no additional support). More than half (55%) of Cambodian students said caregivers were available and able to support learning at home either “often” or “all of the time.”

### 4.5 Impact on basic necessities such as food, income, employment, housing

The data indicated that the COVID-19 pandemic had a substantial negative impact, for both the Australian and Cambodian participants, on basic necessities such as food, income, employment, and housing (refer to Tables 18–21). The questions asked, however, were somewhat different. The UNICEF-Australia survey asked about worries and actual experience regarding family income and own housing, and worries about own income and employment. The UNICEF-Cambodia survey asked about family food access and government financial support (refer to Tables 18–21).

**Table 18:** Things most worried about in relation to the pandemic and responses (Australia).

	***N***	***%***
Losing my housing	81	6.3
My own income and employment being cut back or stopped	185	14.4
Losing our family's income through job loss	398	30.9

**Table 19:** Things experienced since Australia started to respond to the pandemic (Australia).

	***N***	**%**
My parent/s or carer has lost all of part of their income	370	28.7
I have lost my housing/accommodation	41	3.2

**Table 20:** Family experienced change in access to food during school closure (Cambodia).

	***N***	**%**
More quantity	244	8.2
Same quantity	1,940	65.0
Less quantity	782	26.2
Don't know	17	0.6

**Table 21:** Anyone in household received financial support from the government as part of COVID-19 cash assistance scheme or through other private sources (Cambodia).

	***N***	**%**
Yes—from government's cash scheme	255	38.6
Yes—from a private sector cash scheme	37	5.6
Yes—from a local NGO/INGO cash scheme	47	7.1
No	321	48.6

Interestingly, in the UNICEF-Australia survey, responses for worry were not much greater than responses for experience, with 30.9% of participants being worried about losing their family's income through job loss and 28.7% actually experiencing this. Similarly, 6.3% of respondents indicated worry about losing their own housing and 3.2 actually experienced this. Another question in the UNICEF-Australia survey discussed in greater detail in the next section focused on change in household responsibilities (refer to Table 22), including the response option, “I am now the main breadwinner in my family” (chosen by 6.5% of participants), suggesting that one or more parents/carers had lost their job as a result of COVID-19.

**Table 22:** How the COVID-19 pandemic and responses has affected responsibilities at home (Australia).

	***N***	**%**
I do more cooking at home	317	24.6
I do more household maintenance	327	25.4
I do more home cleaning	464	36.0
I look after / supervise my sibling/s more	249	19.3
I have more caring responsibilities for my extended family	176	13.7
I feel a responsibility to pick up as much paid work as I can to help my family income	185	14.4
I am now the main breadwinner in my family	84	6.5
My responsibilities have generally decreased	27	2.1
My responsibilities have not really changed	513	39.8

In response to the UNICEF-Cambodia food access question, 26.2% of respondents said they experienced access to less quantity of food during school closure (while 8% said they had access to a greater quantity). Just over half (51.4%) of the participants who responded to the financial support question said they had received financial support during the pandemic, with most (38.6%) receiving government support. Another question asked in the UNICEF-Cambodia survey, discussed in greater detail in the following section, asked about change in employment and increased contribution to household chores “in order to mitigate the effects of household income decline during COVID-19” (refer to Table 23). Interpretation of this data, however, was difficult. Given the question wording, anyone who either did not experience household income decline or did experience household income decline but did not start working or contributing more to household chores to mitigate its effects should have answered “no.” Therefore, technically, we should be able to conclude that 91.8% of respondents (100%—the 8.2% who said “no”) experienced a decline in household income. Such “double-barreled” survey questions, however, are confusing to respondents and difficult to answer correctly [(43), p. 857–859]. However, given the large percentage of participants (81.7%) who said they started contributing more to household chores in response to household income decline, it can probably be assumed that, for many participants, their household income declined due to COVID-19.

**Table 23:** Started working or contributing more to household chores in order to mitigate the effects of household income decline during COVID-19 (Cambodia).

	***N***	**%**
Yes—working full-time	163	5.5
Yes—working part-time	132	4.5
Yes—contributing more to household chores	2,416	81.7
No	247	8.4

### 4.6 Responsibilities at home

It also appears that participants took on more responsibilities at home due to the COVID-19 pandemic, particularly Cambodian participants. Whereas 82% of Cambodian participants said they were contributing more to household chores (in order to mitigate the effects of household income decline), this compares with 58% of Australian participants (100%—the 39.8% who said their responsibilities have not really changed and the 2.1% who said their responsibilities have decreased). Whether increased responsibilities had a negative or positive impact on participants' mental health and wellbeing, however, is unknown. As discussed above, the UNICEF-Cambodia question on this topic is problematic.

## 5 Discussion

Overall, we found that the impact of COVID-19 early in the pandemic on adolescents' mental health and wellbeing was mostly negative, in both Australia and Cambodia. This is an important finding, given that both surveys were conducted only 4–5 months after the start of the pandemic. This suggests that even short-term disasters may have negative repercussions on the mental health and wellbeing of young people, and despite differences in wealth, culture, and government responses. This overall conclusion accords with most of the literature on this topic, which also finds generally negative impacts of the COVID-19 pandemic on young people's mental health and wellbeing (44–49).

Most of the survey questions in this study, however, assumed neutral or negative impact and did not ask about possible positive impact. Exceptions to this were the UNICEF-Australia questions about overall ability to cope before COVID-19 and currently; effect of Australia's response to the pandemic, including school closures, on schooling, which included a response option that the student was now ahead in their schooling; and impact on responsibilities at home, which included a response option that they have generally decreased. In the UNICEF-Cambodia survey, participants were asked about whether their family had experienced a change in access to food during school closure, with a response option that access had increased. In each case, negative impact was greater than positive impact; nevertheless, it is important to note that there was some positive impact. Existing research has also noted some positive impacts of the COVID-19 pandemic for children or young people, such as greater capacity for self-care and reflection due to decreased pressures of daily life (50, 51); stronger connection/more time with family (33, 52) and greater appreciation of life and death, reorganization of priorities, and strengthened connectedness to community (52). While not downplaying the main finding of our study—that COVID-19 had mostly negative effects on adolescents' mental health and wellbeing in Australia and Cambodia, it would be worthwhile in future research to better understand these possible positive impacts, including why and for whom. This may include utilization of a resilience framework that recognizes the complex interactions between individual, community, and cultural contexts [see, for example Kaye-Kauderer et al. (53)].

Acknowledging the difficulties in comparing countries using results from different surveys with different survey questions, it appears that the negative impacts of the pandemic on personal safety were greater for Cambodian participants than for Australian participants. Although it is unknown what participants in the UNICEF-Australia survey considered “threats to my personal safety,” the fact that 13.9% of Australian participants said that their worry about threats to personal safety had increased since the start of the pandemic, whereas 23.9% of Cambodian participants reported experiencing an increased threat of one or more of the following: violent discipline/physical abuse, sexual abuse or exploitation, emotional abuse (e.g. neglect); child marriage; risk of juvenile offense; receiving a sexual message, inappropriate image or video, or child labor, suggests that the negative impact was greater for Cambodian adolescents.

Why this may be the case is unclear. It may have to do with the higher rate of violence against children in Cambodia that existed pre-COVID, suggesting that such violence is more common in Cambodia than in Australia. As a result of the stress of the pandemic, this rate may have increased more easily. A 2013 cross-sectional household survey of violence against children conducted by the Government of Cambodia found that over three-quarters of children surveyed reported more than one incident of physical violence, emotional violence, or sexual abuse prior to age 18 (https://violenceagainstchildren.un.org/sites/violenceagainstchildren.un.org/files/documents/political_declarations/east_asia_and_pacific/cambodias_violence_against_children_survey.pdf). While Australia has no equivalent statistics on violence against children (54), most estimates are considerably lower than the Cambodian finding (55). In addition, the particularly strong link in Cambodia between school attendance and child safety and wellbeing (56) may have meant that when schools closed, violence against children increased.

Another important issue in comparing the Australian and Cambodian survey results is the terminology used in the surveys to ask about mental health, as well as levels of normalization, stigma, and knowledge around mental health problems in the two countries. In the UNICEF-Cambodia survey, participants were asked whether they had “displayed signs of mental health distress,” whereas in the UNICEF-Australia survey, participants were asked about “ability to cope,” things “most worried about,” and things “experienced.” The use of the term, “mental health distress” may have been unfamiliar to Cambodian participants, as it is a more technical term and one borrowed from Western countries (57). This potential lack of clarity may have affected responses (58).

Level of stigma attached to mental health issues may be greater in Cambodia than in Australia, with the term, “crazy,” still commonly used to describe those struggling with mental illness, and with very low rates of help seeking (59–61). Levels of stigma around mental illness in Australia, however, while improving, continue to be an issue, with stereotypical attitudes, discrimination, exclusion, and the perception that people with mental illness are dangerous, unpredictable, and lack competence to take care of themselves persisting (62).

Mental health literacy appears to be lower in Cambodia than in Australia (63). A study comparing scores on a mental health literacy assessment between Cambodian and Australian students and found that the Australian students scored significantly higher (64). These between-country differences may have resulted in suppressed responses amongst Cambodian participants. Therefore, it may be that the impact of COVID-19 on the mental health of Cambodian young people was greater than these survey results suggest.

Support for online or distance schooling during periods of school shutdowns was considered inadequate by large percentages of survey participants in both Australia and Cambodia—particularly support from schools or the education system, as compared with support from parents or carers (65). Much has been written about the challenges faced by schools in providing an adequate virtual educational experience for students during school closures, including lack of preparedness to transition to remote or online learning, poor ICT infrastructure, lack of technology skills of teachers, and difficulties reaching students and parents (66–68). At the same time, parents and carers also struggled to provide adequate support for their children, with issues such as time, capability, and the ability to provide a home environment conducive to online study (69–72). These findings indicate that the education systems in both countries were unprepared to effectively respond to this type of disruption, and that plans must be developed and enacted to be ready for the next disruption.

As with any study, this one had its strengths and limitations. Limitations included that the surveys used to gather the data asked different questions and used different methodologies and that no standardized measures of mental health and wellbeing were used. Other limitations were the cross-sectional design, which precludes causal interpretations; descriptive analyses; and lack of comparable pre-pandemic data and subgroup analyses. Also, effects on physical health were not considered (73). Strengths included the use of national data from both countries, gathered from the same population (adolescents) on approximately the same dates on many of the same subjects by the same organization (UNICEF).

The most obvious recommendation for future research is a single survey administered in both (and additional) countries. In addition, qualitative research would be helpful to provide greater context and understanding of the quantitative data.

## 6 Conclusion

This study examined the early impact of the COVID-19 pandemic on the mental health and wellbeing of adolescents in two countries—one developed (Australia) and one developing (Cambodia)—through a descriptive and comparative approach using two different surveys conducted with similar populations on similar topics at a similar time.

It found that COVID-19 had mostly negative impacts on participants' mental health and wellbeing, including their mental health; threats to personal safety; education; support for schooling; basic necessities such as food, income, employment, and housing; and responsibilities at home. It also found that there were some positive impacts for some young people and suggested that these positive impacts should be further explored and better understood. It found that threats to personal safety appeared to be more widespread in Cambodia than in Australia. It also surmised that, due to both question wording and a greater level of stigma around mental health illness in Cambodia, the impact on mental health of the Cambodian participants may have been greater than reported. Finally, it found that, in both countries, support for online or distance schooling during periods of lockdown was lacking particularly at the state and school levels.

It is hoped that this study will add to the evidence base on the impact of major disruptive events on adolescents in both economically developed and less economically developed countries. The main findings also have potential clinical implications in terms of interventions aimed at reducing the impact of COVID-19 or other major disruptive events on the mental health and wellbeing of adolescents in Australia and Cambodia, and potentially other countries.

## Data availability statement

The data analyzed in this study was obtained from UNICEF-Cambodia and UNICEF-Australia. Requests to access these datasets should be directed to Linda Jonsson, ljonsson@unicef.org (UNICEF-Camodia) and Nicole Breeze, nbreeze@UNICEF.org.au (UNICEF-Australia).

## Ethics statement

All UNICEF commissioned surveys must adhere to the UNICEF Procedures for Ethical Standards in Research, Evaluation, Data collection, and Analysis without going through a formal Human Research Ethics procedure (39). In both countries, informed consent from participants was obtained. No identifying information was included in either data set used for this analysis. The studies were conducted in accordance with the local legislation and institutional requirements. The participants provided their written informed consent to participate in this study.

## Author contributions

NV: Conceptualization, Data curation, Formal analysis, Investigation, Methodology, Writing – original draft, Writing – review & editing. SK: Data curation, Formal analysis, Investigation, Writing – review & editing. MC: Conceptualization, Writing – review & editing.
